# Antineuropathic and Antinociceptive Drugs Combination in Patients with Chronic Low Back Pain: A Systematic Review

**DOI:** 10.1155/2012/154781

**Published:** 2012-04-26

**Authors:** Carlo Luca Romanò, Delia Romanò, Marco Lacerenza

**Affiliations:** ^1^Centro di Chirurgia Ricostruttiva, Istituto Ortopedico I.R.C.C.S. Galeazzi, Via R. Galeazzi 4, 20161 Milano, Italy; ^2^Centro di Medicina del Dolore, Casa di cura S. Pio X, Fondazione Opera San Camillo, Via F. Nava 31, 20159 Milano, Italy

## Abstract

*Purpose*. Chronic low back pain (LBP) is often characterized by both nociceptive and neuropathic components. While various monotherapies have been reported of only limited efficacy, combining drugs with different mechanisms of action and targets appears a rational approach. Aim of this systematic review is to assess the efficacy and safety of different combined pharmacological treatments, compared to monotherapy or placebo, for the pharmacological treatment of chronic LBP. *Methods*. Published papers, written or abstracted in English from 1990 through 2011, comparing combined pharmacological treatments of chronic LBP to monotherapy or placebo were reviewed. *Results*. Six articles met the inclusion criteria. Pregabalin combined with celecoxib or opioids was shown to be more effective than either monotherapy. Oxycodone-paracetamol versus previous treatments and tramadol-paracetamol versus placebo were also reported as effective, while morphine-nortriptyline did not show any benefit over any single agent. *Conclusions*. In spite of theoretical advantages of combined pharmacological treatments of chronic LBP, clinical studies are remarkably few. Available data show that combined therapy, including antinociceptive and antineuropathic agents is more effective than monotherapy, with similar side effects.

## 1. Introduction

Successful treatment of chronic pain depends on identification of the involved mechanism and use of appropriate therapeutic approaches. Woolf et al. [1] proposed that pain symptoms and syndromes should be classified into two broad mechanism-based pain categories: tissue-injury pain (nociceptive) or nervous-system-injury pain (neuropathic).

Even if there is increasing knowledge that different mechanisms of pain require appropriate treatments and often polypharmacotherapy, and although drug combination is frequently empirically adopted in the clinical practice [2–5], prospective studies concerning the relative efficacy and safety of therapeutical drug associations to treat various painful conditions are still remarkably few [6–10] and, as recently reported, “*more preclinical, clinical, and translational studies are needed to improve the efficacy of combination drug therapy that is an integral part of a comprehensive approach to the management of chronic pain*” [11].

Although many patients have self-limited episodes of acute low-back pain (LBP) and do not seek medical care [12], this condition is among the five most common reasons for all physician visits in the USA [13, 14]. Among those who do seek medical care, pain, disability, and return to work typically improve rapidly in the first month [15]; however, up to one-third of patients report persistent back pain of at least moderate intensity one year after an acute episode [16, 17].

Medications are the most frequently recommended intervention for low back pain [14, 18]. In one study, 80% of primary care patients with low back pain were prescribed at least one medication at their initial office visit, and more than one-third were prescribed two or more drugs [5]. The most commonly prescribed medications for low back pain are nonsteroidal anti-inflammatory drugs (NSAIDs), skeletal muscle relaxants, and opioid analgesics [5, 19, 20]. Benzodiazepines, systemic corticosteroids, antidepressant medications, and antiepileptic drugs are also prescribed [21]. Monotherapies of chronic LBP with NSAIDs, acetaminophen and tricyclic antidepressants, opioids, tramadol, benzodiazepines, and gabapentin (for radiculopathy) have all been found to provide only a limited pain relief, ranging from 10 to 20 points on a 100-point visual analogue pain scale [22].

Chronic LBP has been shown to be the result of neuropathic as well as nociceptive pain mechanisms and has therefore been classified as a mixed pain syndrome [23–25]. Nonspecific nociceptive pain is the result of an inflammatory response to tissue injury, while neuropathic pain describes cutaneous projected pain arising from the lumbar spine and/or nerve roots (radicular pain or radiculopathy) [3, 4]. The multifactorial nature of chronic LBP has often been underrecognized and undertreated. Thus, recent studies have demonstrated that approximately 20–55% of patients with chronic LBP have a >90% likelihood of a neuropathic pain component and, in an additional 28% of patients, a neuropathic pain component is suspected [5, 26, 27]. The presence of a neuropathic pain component is associated with more severe pain symptoms and higher healthcare utilization costs [28].

Based on this evidence, it has been suggested that antidepressants and/or anticonvulsants in combination with either opioids, traditional nonsteroidal anti-inflammatory drugs, or muscle relaxants could be useful in the treatment of this condition [27, 29, 30]. The aim of this systematic review is to evaluate evidence for the effectiveness of pharmacological combination therapy in chronic LBP, with specific reference to the management of nociceptive and neuropathic pain components.

## 2. Materials and Methods

Published papers written in English or including an English abstract, published from 1990 through 2011 and reporting the results of a combined pharmacological treatment of chronic lowback pain (LPB), compared with monotherapy or placebo, were reviewed. To this aim, we searched international databases, including EMBASE, PubMed/Medline, Google Scholar, SCOPUS, CINAHL, Cochrane Central Register of Controlled Trials, Cochrane Database of Systematic Reviews, http://www.google.com/, and http://www.yahoo.com/. Inclusion criteria were the following:

- papers written or with an abstract in English;
- papers concerning the results of management of chronic low-back pain (symptoms duration >6 months);
- treatment using a combination of two or more drugs, versus monotherapy or placebo.

Two investigators, CR and ML, searched and reviewed independently the literature and classified the references found in terms of whether they should be included on basis of the title and the abstract of the paper. In addition to original study reports, review articles were also included and the reference lists from all reviewed articles were assessed to complete the literature search. At the end of the reviewing process, the two reviewers' lists of papers were compared and if any discrepancy occurred, reclassification was performed according to the consensus reached.

**Figure 1:**
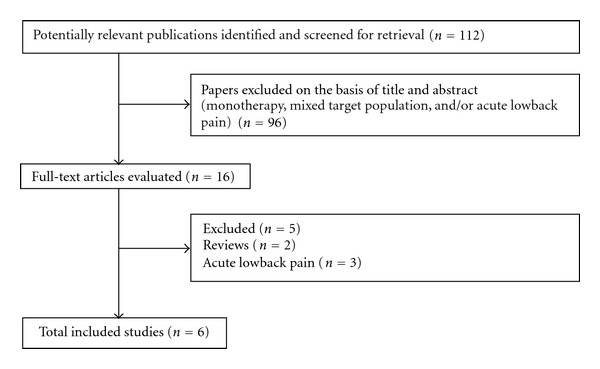
Flow diagram of inclusion and exclusion of articles for combined pharmacological interventions for chronic low back pain.

This strategy identified 112 articles, the abstracts of which were hand searched to identify a subset with the specific focus of pharmacological treatment of chronic LBP of relevance to the current review. Six studies on pharmacological management of chronic LBP (irrespective of the cause) were identified as relevant and were included in this paper (Figure 1).

## 3. Results

Table 1 summarizes the included studies examining combination pharmacotherapy of chronic LBP. Three studies evaluated paracetamol in combination with tramadol [35, 36] or oxycodone [32].

In the first study (*n* = 318), three-month treatment with tramadol 37.5 mg/paracetamol 325 mg yielded significantly greater improvements in pain VAS score (*P* < 0.015) and Pain Relief Rating Scale score (*P* < 0.001) than placebo. Significant improvements were also observed for Roland Disability Questionnaire (RDQ) scores, several of the sensory Short-Form McGill Pain Questionnaire (SF-MPQ) items, and the Role-Physical, Bodily Pain, Role-Emotional, Mental Health, Reported Health Transition and Mental Component items of the Short Form 36 (SF 36; all *P* < 0.05). The rates of discontinuation due to insufficient pain relief were significantly lower with tramadol plus paracetamol (22.1%) than for placebo (41.0%; *P* < 0.001), and the proportion of patients and investigators rating treatment as “good” or “very good” was higher with combination therapy than with placebo (*P* < 0.001 for patients; *P* = 0.002 for investigators). Adverse events were more common with the combination (68.9%) than with placebo (46.5%), as were adverse drug reactions (23.6% versus 3.8%) and rates of discontinuation due to adverse events (18.6% versus 5.7%). Nausea, somnolence, and constipation were significantly more frequent with combination treatment than with placebo (*P* < 0.05) [36].

In the second study, patients with at least moderate chronic LBP received tramadol 37.5 mg/paracetamol 325 mg in a fixed combination tablet; VAS scores after 3 months were significantly lower with tramadol/paracetamol than with placebo (*P* < 0.001). Combination therapy was also associated with significantly improved scores on several measures, including RDQ score and physical-related items on the SF-MPQ and SF-36 (*P* < 0.05). Similar results to those reported above by Ruoff et al. [36] were observed for discontinuation due to insufficient pain relief, the proportion of patients rating treatment as “good” or “very good” and the incidence of adverse events [35].

Gatti et al., in a prospective observational study [32] examined the efficacy of a fixed-dose combination of oxycodone plus paracetamol using the Pain Management Index. Patients were stratified according to the presence of prevalent osteoarticular pain (*n* = 78) or prevalent neuropathic pain (*n* = 72). Combination therapy was associated with an improvement in pain in the majority of compliant patients, although its benefit in patients with neuropathic pain was less marked.

Two papers reported on the efficacy of pregabalin with, respectively, celecoxib [31] or transdermal (TDS) buprenorphine [33]. In our previously published prospective, single-blind, randomized study [31], the safety and efficacy of the association of celecoxib and pregabalin with either monotherapy for treatment of chronic low back pain of various origin, were compared; data were also analyzed on the basis of pain quality assessed with the Leeds Assessment of Neuropathic Symptoms and Signs (LANSS) pain scale [37, 38]. Our study showed that the association pregabalin/celecoxib resulted in a statistically significant reduction of self-reported pain when considering either all the recruited patients or the subpopulations divided according to LANSS score. On the contrary, celecoxib/placebo and pregabalin/placebo only produced a statistically significant reduction of reported pain in, respectively, patients with LANSS score <12 (*P* = 0.01) (nociceptive pain) and in patients with LANSS score >12 (*P* = 0.03) (neuropathic pain), but not when including all the patients. The drug combination also proved to be more effective than pregabalin alone or than celecoxib alone, except for patients with LANSS score <12, in which treatment combination or monotherapy provided similar results. When all patients were considered, celecoxib alone provided 12.4% pain reduction, pregabalin alone 10.4%, and their combination 38.2%. The largest pain reduction (51.8%) was observed with the association pregabalin/celecoxib in patients with LANSS score > 12. Pregabalin drug consumption, when used in association with celecoxib, was significantly lower (*P* < 0.05) compared to monotherapy. The occurrence of side effects was similar during either monotherapy or combination treatment [31].

Similarly, Pota and coworkers [33] found that the combination of pregabalin and buprenorphine, TDS yielded significantly greater reductions in VAS scores than buprenorphine monotherapy (*P* < 0.01). In the first month of therapy buprenorphine TDS alone provided a meaningful pain reduction (VAS 82.75 ± 15 versus 38.25 ± 5, *P* < 0.01); at the end of the first month, patients were then divided in two groups: Group A receiving one-month therapy with buprenorphine 35 *μ*g/mL plus pregabalin 150 mg and Group B buprenorphine plus placebo. At the end of the treatment, only Group A presented a further reduction of the VAS (*P* < 0.01). The authors concluded that “*buprenorphine TDS determines a notable relief from pain. Moreover the association of low doses of pregabalin allowed a further relief.*”

The unique study evaluating the combination of morphine with nortriptyline [34], failed to provide sufficient data as to regard the efficacy of this free opioid-antidepressant combination for the treatment of chronic LBP. In this study, performed on 61 patients with sciatica, the combination of morphine and nortriptyline did not reduce average leg pain scores or any other leg or back pain scores, while 89% of patients receiving combination treatment reported an adverse event, most commonly constipation.

## 4. Discussion

This systematic review shows that, in spite chronic LBP is thought to be commonly the result of both nociceptive and neuropathic mechanisms [23] and hence a rationale approach would be targeting the different mechanisms of pain by combining specific drug agents, remarkably few clinical trials are currently available to validate this hypothesis.

This may due to different reasons including

- the difficulty in designing/performing clinical trials involving more treatments at the same time;
- potential drugs' interactions and possible adverse effects. Any specific combination of agents need to be first evaluated on the basis of the respective pharmacokinetic profile and possible interactions and then clinically tested. In free dose combinations, the onset of adverse events can, to some extent, be overcome by initiating treatment at low doses and slowly escalating the dose until maximum analgesia or intolerable side effects arise; drug combination may also provide reduced consumption of any single drug and adverse events comparable to monotherapy [31];
- unpredictable dosing regimen. At variance with that reported above concerning the possible advantage of free dose combinations, fixed dose are easier to study and to market, being also likely associated with greater patient's compliance than free combinations; however, identifying the best dose ratio for all the patients, balancing the efficacy and tolerability of any single drug within a fixed combination may be a challenging exercise;
- scarce economical interest of drug companies.

All of these potential drawbacks may, to a different extent, concur to explain the limited research on drug combinations, in spite of theoretical positive considerations and notwithstanding the empirical widespread use of drug associations in the clinical practice [5].

Among the six studies that were included in the present review, two examined a fixed-dose regimen of paracetamol and tramadol combination against placebo [35, 36]. These studies appear much similar, in their design and outcomes, to a “traditional” monotherapy versus placebo study [39] and do not really seem to add any insight as to concern the control of different types of pain.

On the contrary, the association of pregabalin plus celecoxib [31] or of pregabalin and an opioid agent [33] seem more focused on targeting different pain components of chronic LBP.

Gabapentinoids have already been successfully used in combination with other analgesic drugs to improve neuropathic pain control. Gilron et al. [7] first reported on the efficacy and safety of a combination of gabapentin and morphine compared with that of each as a single agent in patients with painful diabetic neuropathy or postherpetic neuralgia. In 41 patients, gabapentin-morphine combination showed significantly better pain control (*P* < 0.05) versus placebo, gabapentin, and morphine. More recently, Gatti et al. reported the Multicenter Italian Study, which compared the efficacy, safety, and quality of life of combination therapy with controlled release (CR) oxycodone plus pregabalin versus monotherapy in patients with neuropathic pain of various origins [40]. This study showed in 409 patients that the combination of CR oxycodone plus pregabalin was more effective than monotherapy for alleviating neuropathic pain (*P* = 0.003) and to improve quality of life (*P* = 0.0009), while combination therapy also allowed dose reduction of both agents (22% for CR oxycodone and 51% for pregabalin).

Interestingly, in our reported study, celecoxib or pregabalin when used alone were shown to be not effective in patients with, respectively, neuropathic or nociceptive low back pain type, as evaluated with the LANSS pain scale [31]. This is not surprising, given the specific ability of pregabalin to control neuropathic pain [2, 41, 42], while celecoxib is a selective COX-2 inhibitor that has been proved to be effective in the treatment of different pain models that are considered predominantly of nociceptive origin [43, 44]. However, this finding also supports the hypothesis of a better efficacy of a combined approach to the mixed pain conditions and points out the importance of patient selection when evaluating the analgesic efficacy of any specific treatment.

A recent systematic review of pharmacological monotherapies for chronic nonspecific low back pain [45] showed no effects of different types of antidepressants, compared to placebo, on any of the primary investigated outcomes, including pain intensity, depression and functional status. The study from Khoromi et al. [34], in a mixed pain population, suffering from low back pain with lumbar radiculopathy, seems to confirm, with the limitation imposed by the small sample size, that even nortriptyline alone or in combination with morphine has limited effectiveness.

Other frequently prescribed medications, like muscle relaxants [19, 20], have not been investigated in randomized clinical trials for the treatment of chronic low-back pain [45] and we could not find any study regarding their use in a combined pharmacological therapy of this condition.

It is worth noting how published comprehensive reviews of clinical trials [46] and even the most recently reported guidelines concerning the treatment of chronic low back pain fail to address the use of combined pharmacological treatments [47, 48]. While, in fact, several drugs are compared and recommended as monotherapy, associations are not mentioned. The paucity of the available data may well-explain, in our opinion, the lack of indications in this regard and points out the need for further research and well designed clinical trials.

## 5. Conclusions

Pain treatment should be guided by the underlying mechanisms and should take into consideration pain quality as well as pain intensity. Chronic LBP often comprises both nociceptive and neuropathic components, and various monotherapies have been repeatedly reported as only partially effective. Therefore, an individualized, multimodal therapy, combining drugs with different mechanisms of action represents a rational approach. However, available studies investigating drug combinations are remarkably few. In particular, combination of pregabalin and celecoxib or buprenorphine has been demonstrated to be more effective that either monotherapy and relatively safe. The association of paracetamol with tramadol or oxycodone has also been shown to be effective for reducing chronic low-back pain, even if not evaluated against respective monotherapy. Further research in combined pharmacological treatment of chronic LBP with well-designed studies may offer valuable tools for the clinical practice and is strongly suggested.

## Figures and Tables

**Table 1:** Table 1 Clinical trials on combination pharmacological therapy of chronic low back pain.

Reference	Trial design	Duration	Main inclusion/exclusion criteria	Pain type	Intervention(s) and dose	Principal outcome
L. Romanò et al. [31]	Prospective, randomized, 3-way cross-over study 4-weeks treatment with each therapy	12 week	18–75 years Chronic LBP for >6 months due to disc prolapse, lumbar spondylosis, and/or spinal stenosis Minimum VAS >40 mm (on a scale of 0–100 mm) Patients with neurological disease excluded	Mixed	Celecoxib 3–6 mg/kg/die + placebo (*n* = 36) and Pregabalin 1 mg/kg/die for the first week, then 2–4 mg/kg/die + placebo (*n* = 36) and Celecoxib 3–6 mg/kg/die + pregabalin 1 mg/kg/die for the first week, then 2–4 mg/kg/die (*n* = 36)	Combination therapy was more effective than either monotherapy for mean pain reduction (assessing using 0–100 mm VAS)

Gatti et al., [32]	Prospective, observational, and open-label study	6 week	Chronic LBP (46 months) Moderate to severe (>3 on a 0–10 VAS) Pain not responsive to previous systemic or local analgesic treatment	Osteoarticular, nociceptive pain (Group A) or neuropathic pain (Group B)	Group A Previous treatment discontinued: Oxycodone 5 mg + paracetamol 325 mg/8 hours (*n* = 78) Group B Previous treatment (except gabapentin). Fixed combination of oxycodone 5mg + paracetamol 325 mg/8 hours (*n* = 72)	Group A 73.9% and 78.3% (assessed using 0–10 VAS), respectively Group B All patients reported improved or stable neuropathic pain symptoms except pain preventing sleep

Pota et al., [33]	Prospective, observational, and open-label study	2 month	Chronic LBP (33 months)	Mixed	(*n* = 22) Month 1: Buprenorphine TDS 35 *μ* g/ml Month 2: Buprenorphine TDS 35 *μ* g/ml + pregabalin 150 mg or Buprenorphine TDS 35 *μ* g/ml + placebo	Significant reductions in pain(assessed using 0–100 VAS) were observed after month 1 (*P* < 0.01) Significant reductions in painafter month 2 were only observed in the combination group (*P* < 0.01)

Khoromi et al., [34]	Single-centre, cross-over, randomized trial	9 week	18–65 years Lumbar radiculopathy Average leg pain score >4 (0–10 cm VAS) Patients with polyneuropathy and peripheral vascular disease associated with symptoms of numbness, or patients with burning painin the lower extremities, were excluded	Neuropathic	(*n* = 61) Morphine 15–90 mg and Nortriptyline 25–100 mg and Morphine 15–90 mg nortriptyline 25–100 mg	No significant reductions in mean leg pain (assessed using 0–10 VAS) or other leg or back pain were observed in any treatment group Pain reduction relative to placebo was 14% for nortriptyline, 7% for morphine, and 7% for combination therapy

Peloso et al., [35]	Multi-centre, randomized, double-blind study	21-day washout period, 91-day double-blind treatment period	> 18 years Chronic LBP Pain intensity >40 (0–100 mm VAS) Patients with neurologic deficits in lower extremities, symptomatic disk herniation, severe spinal stenosis, or spondy lolisthesis excluded	Nociceptive	Tramadol 37.5–300 mg + paracetamol 325–2600 mg (*n* = 167) or Placebo (*n* = 169)	Mean final pain intensity scores (assessed using 0–100 mm VAS) were significantly lower with combination therapy (47.4) than with placebo (62.9; <0.001), as were mean final pain relief scores (assessed on 6-point Likertscale: 1.8 and 0.7, resp., *P* < 0.001)

Ruoff et al., [36]	Multi-centre, randomized, double-blind, parallel group study	21-day washout period, 10-day titration period, 81-day treatment period	25–75 years Chronic LBP Pain intensity >40 (0–100 mm VAS)	Mixed	Tramadol 37.5–300 mg + paracetamol 325–2600 mg (*n* = 161) OR Placebo (*n* = 157)	Significantly lower final meanpain score (assessed by 0–100 mm VAS) with combination therapy than with placebo (*P* < 0.015)
CR=controlled release; LBP: low back pain; Mixed: mixed nociceptive and neuropathic pain; TDS: Transdermal delivery system; VAS: visual analogue scale.						
